# Supplementary material for: Ribosomal Dysfunction Is a Common Pathomechanism in Different Forms of Trichothiodystrophy
Source: Cells. 2023 Jul 17;12(14):1877. doi: 10.3390/cells12141877 (PMC10377840; doi:10.3390/cells12141877)
Supplement: Supplementary file 1 [file cells-12-01877-s001.zip › cells-2269489-supplementary.pptx]

## Slide 1
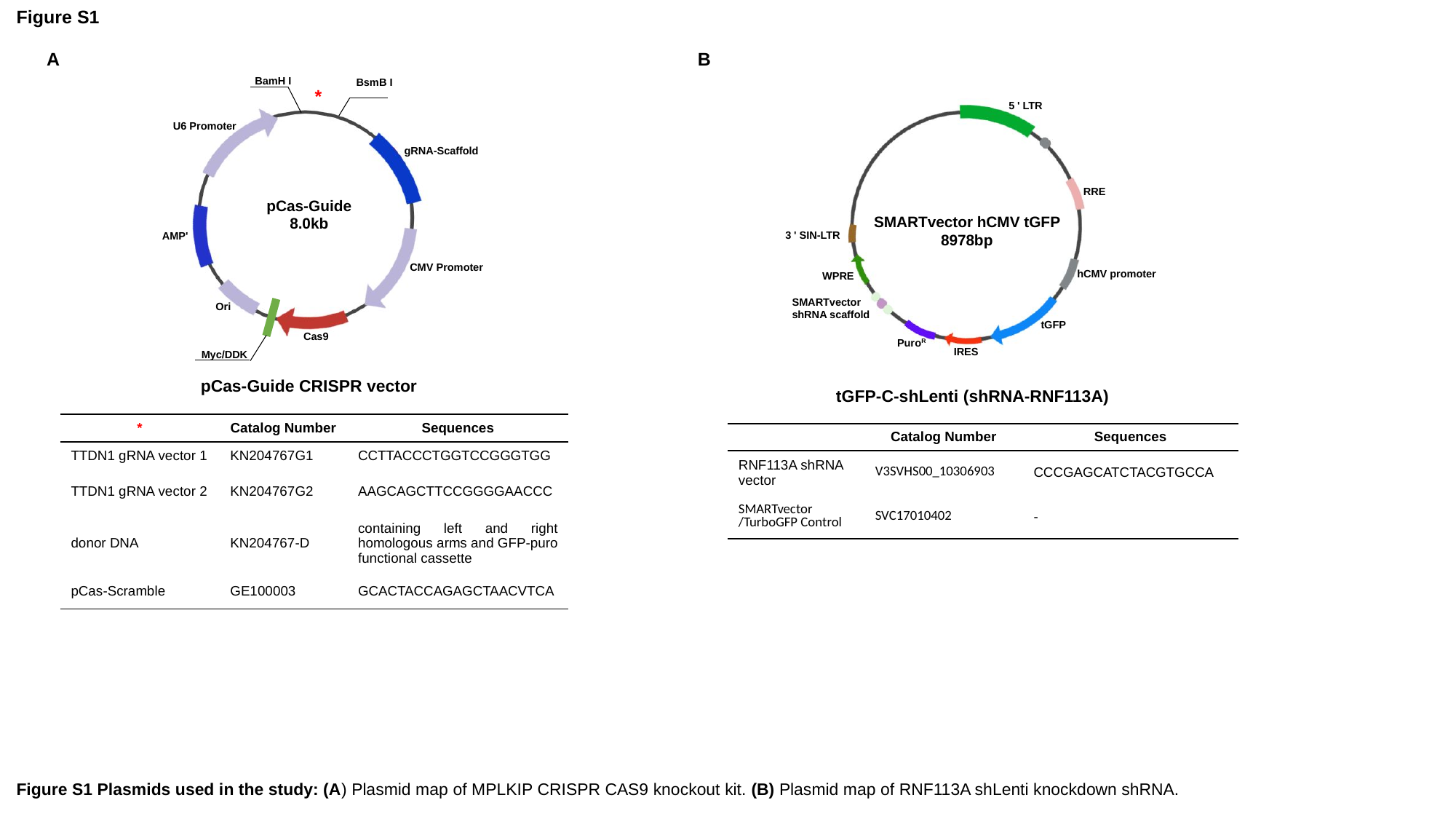

Figure S1
A
B
BamH I
BsmB I
*
U6 Promoter
gRNA-Scaffold
pCas-Guide
8.0kb
AMP'
Ori
Cas9
Myc/DDK
CMV Promoter
5 ' LTR
RRE
SMARTvector hCMV tGFP
8978bp
3 ' SIN-LTR
hCMV promoter
WPRE
SMARTvector shRNA scaffold
tGFP
PuroR
IRES
 pCas-Guide CRISPR vector
tGFP-C-shLenti (shRNA-RNF113A)
| \* | Catalog Number | Sequences |
| --- | --- | --- |
| TTDN1 gRNA vector 1 | KN204767G1 | CCTTACCCTGGTCCGGGTGG |
| TTDN1 gRNA vector 2 | KN204767G2 | AAGCAGCTTCCGGGGAACCC |
| donor DNA | KN204767-D | containing left and right homologous arms and GFP-puro functional cassette |
| pCas-Scramble | GE100003 | GCACTACCAGAGCTAACVTCA |
| | Catalog Number | Sequences |
| --- | --- | --- |
| RNF113A shRNA vector | V3SVHS00\_10306903 | CCCGAGCATCTACGTGCCA |
| SMARTvector /TurboGFP Control | SVC17010402 | - |
Figure S1 Plasmids used in the study: (A) Plasmid map of MPLKIP CRISPR CAS9 knockout kit. (B) Plasmid map of RNF113A shLenti knockdown shRNA.

## Slide 2
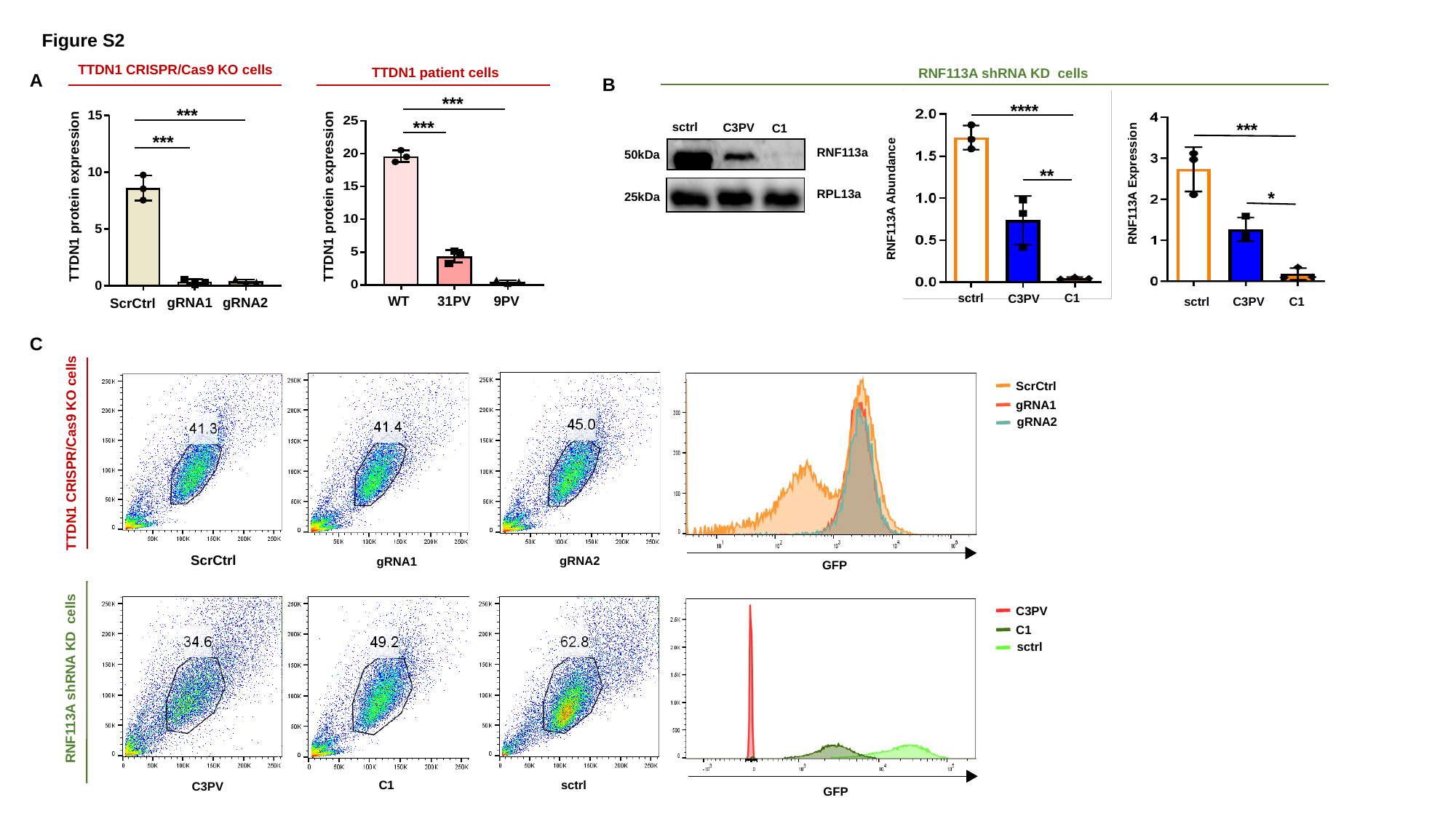

Figure S2
RNF113A Expression
***
*
C3PV
sctrl
C1
RNF113A Abundance
****
**
C1
sctrl
C3PV
TTDN1 CRISPR/Cas9 KO cells
TTDN1 patient cells
RNF113A shRNA KD cells
A
B
***
***
TTDN1 protein expression
31PV
9PV
WT
***
***
TTDN1 protein expression
gRNA2
gRNA1
ScrCtrl
sctrl
C3PV
C1
RNF113a
50kDa
RPL13a
25kDa
C
ScrCtrl
gRNA1
gRNA2
ScrCtrl
gRNA2
gRNA1
GFP
TTDN1 CRISPR/Cas9 KO cells
C3PV
C1
sctrl
RNF113A shRNA KD cells
C1
sctrl
C3PV
GFP

## Slide 3
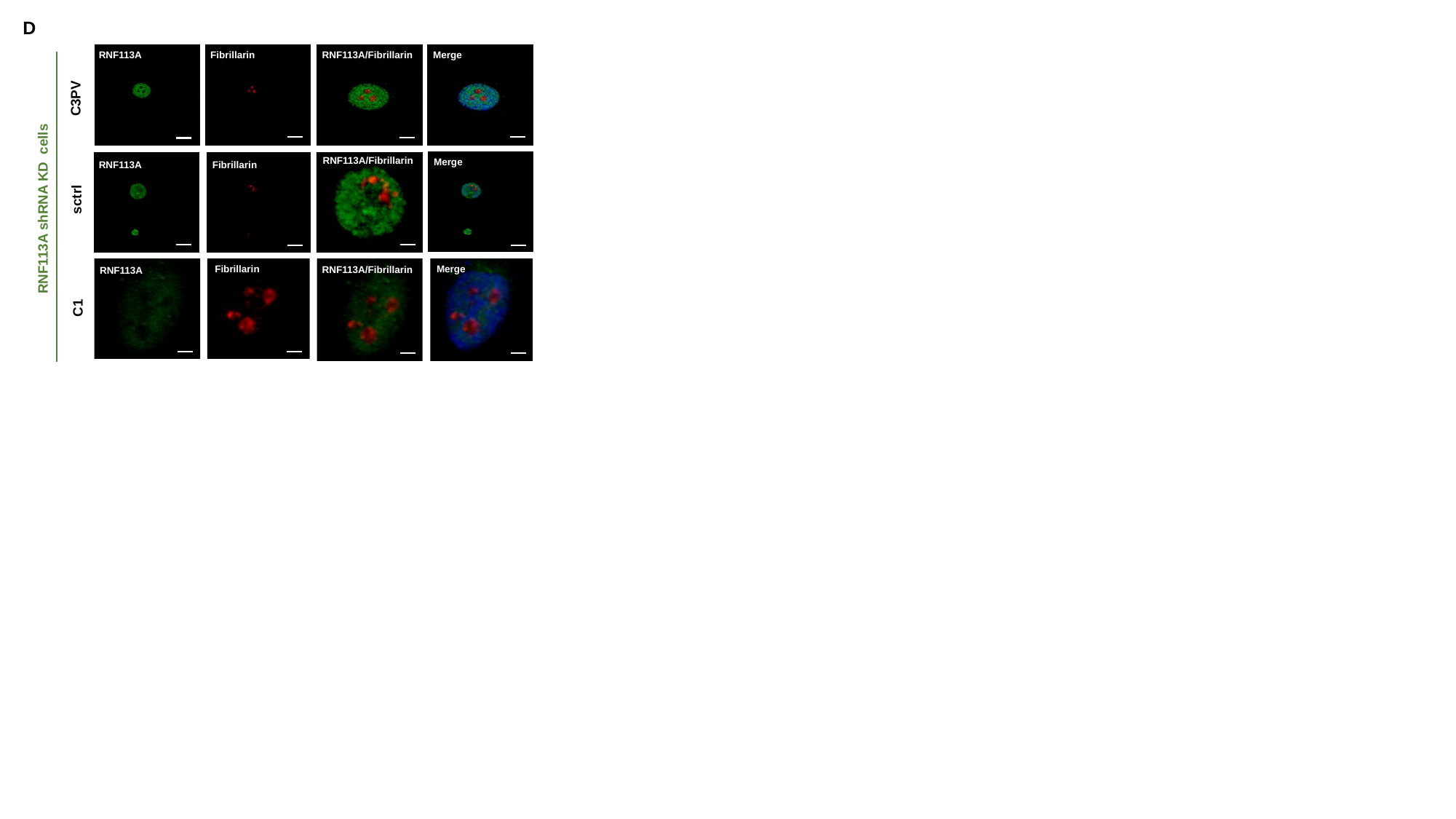

D
Fibrillarin
Merge
RNF113A
RNF113A/Fibrillarin
C3PV
RNF113A/Fibrillarin
Merge
RNF113A
Fibrillarin
sctrl
Fibrillarin
Merge
RNF113A/Fibrillarin
RNF113A
C1
RNF113A shRNA KD cells

## Slide 4
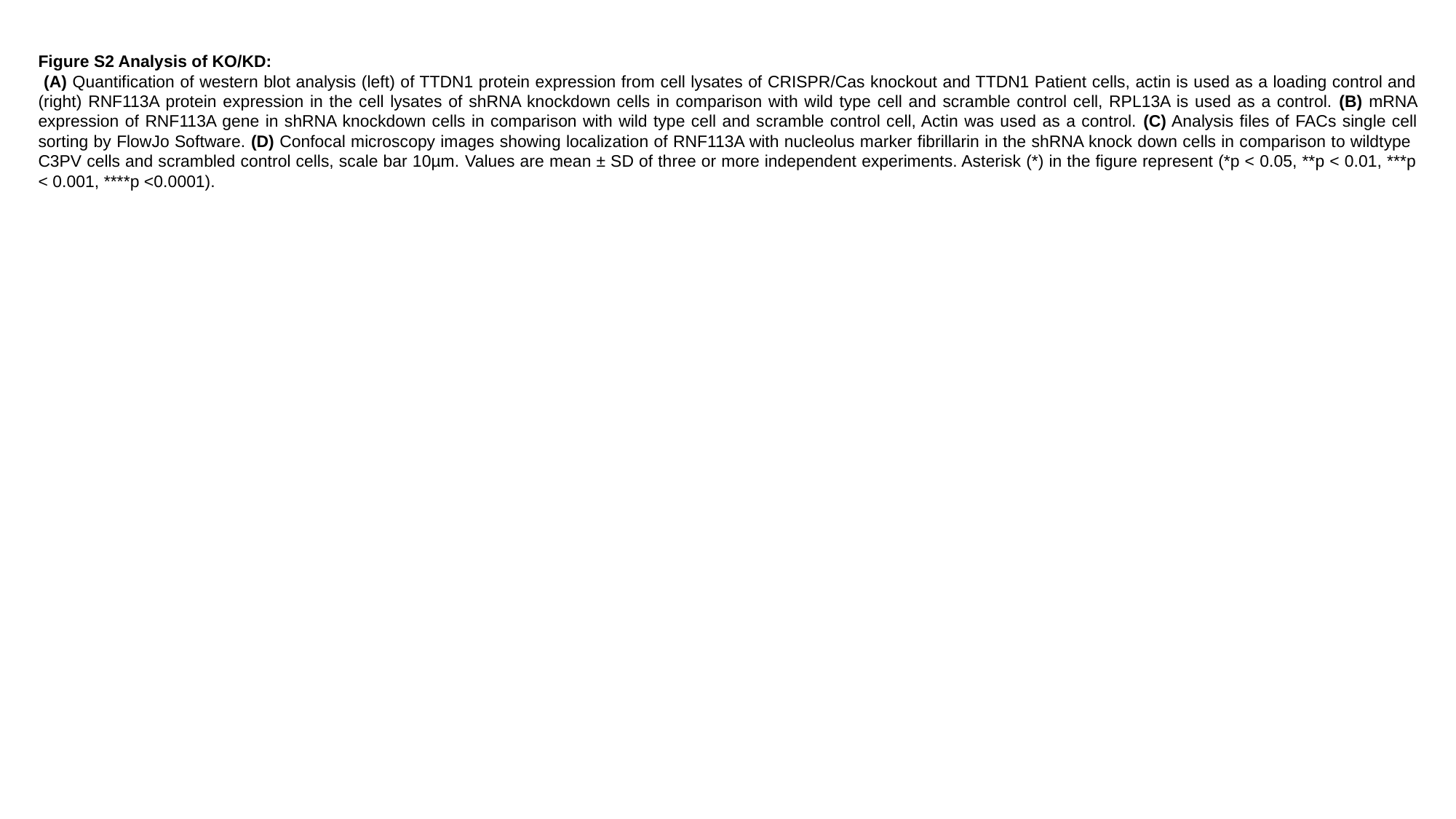

Figure S2 Analysis of KO/KD:
 (A) Quantification of western blot analysis (left) of TTDN1 protein expression from cell lysates of CRISPR/Cas knockout and TTDN1 Patient cells, actin is used as a loading control and (right) RNF113A protein expression in the cell lysates of shRNA knockdown cells in comparison with wild type cell and scramble control cell, RPL13A is used as a control. (B) mRNA expression of RNF113A gene in shRNA knockdown cells in comparison with wild type cell and scramble control cell, Actin was used as a control. (C) Analysis files of FACs single cell sorting by FlowJo Software. (D) Confocal microscopy images showing localization of RNF113A with nucleolus marker fibrillarin in the shRNA knock down cells in comparison to wildtype C3PV cells and scrambled control cells, scale bar 10µm. Values are mean ± SD of three or more independent experiments. Asterisk (*) in the figure represent (*p < 0.05, **p < 0.01, ***p < 0.001, ****p <0.0001).

## Slide 5
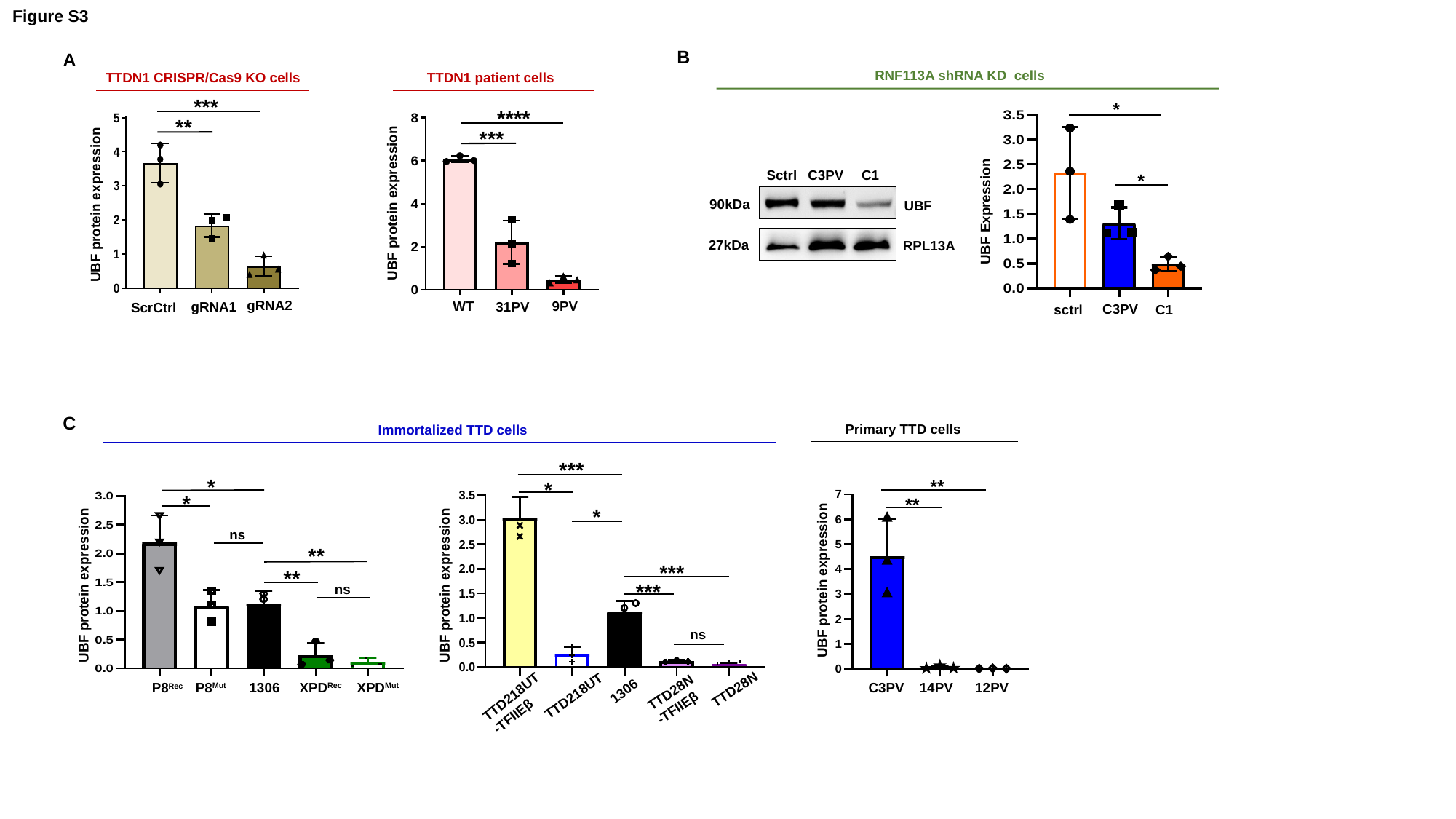

Figure S3
B
A
RNF113A shRNA KD cells
TTDN1 CRISPR/Cas9 KO cells
TTDN1 patient cells
***
**
UBF protein expression
gRNA2
gRNA1
ScrCtrl
*
*
UBF Expression
C3PV
sctrl
C1
****
***
Sctrl
C3PV
C1
90kDa
UBF
27kDa
RPL13A
UBF protein expression
9PV
WT
31PV
C
Primary TTD cells
Immortalized TTD cells
***
UBF protein expression
TTD28N
1306
TTD28N
-TFIIEβ
TTD218UT
TTD218UT
-TFIIEβ
*
*
***
***
ns
*
*
ns
**
**
UBF protein expression
ns
XPDMut
1306
P8Mut
XPDRec
P8Rec
**
**
UBF protein expression
C3PV
14PV
12PV

## Slide 6
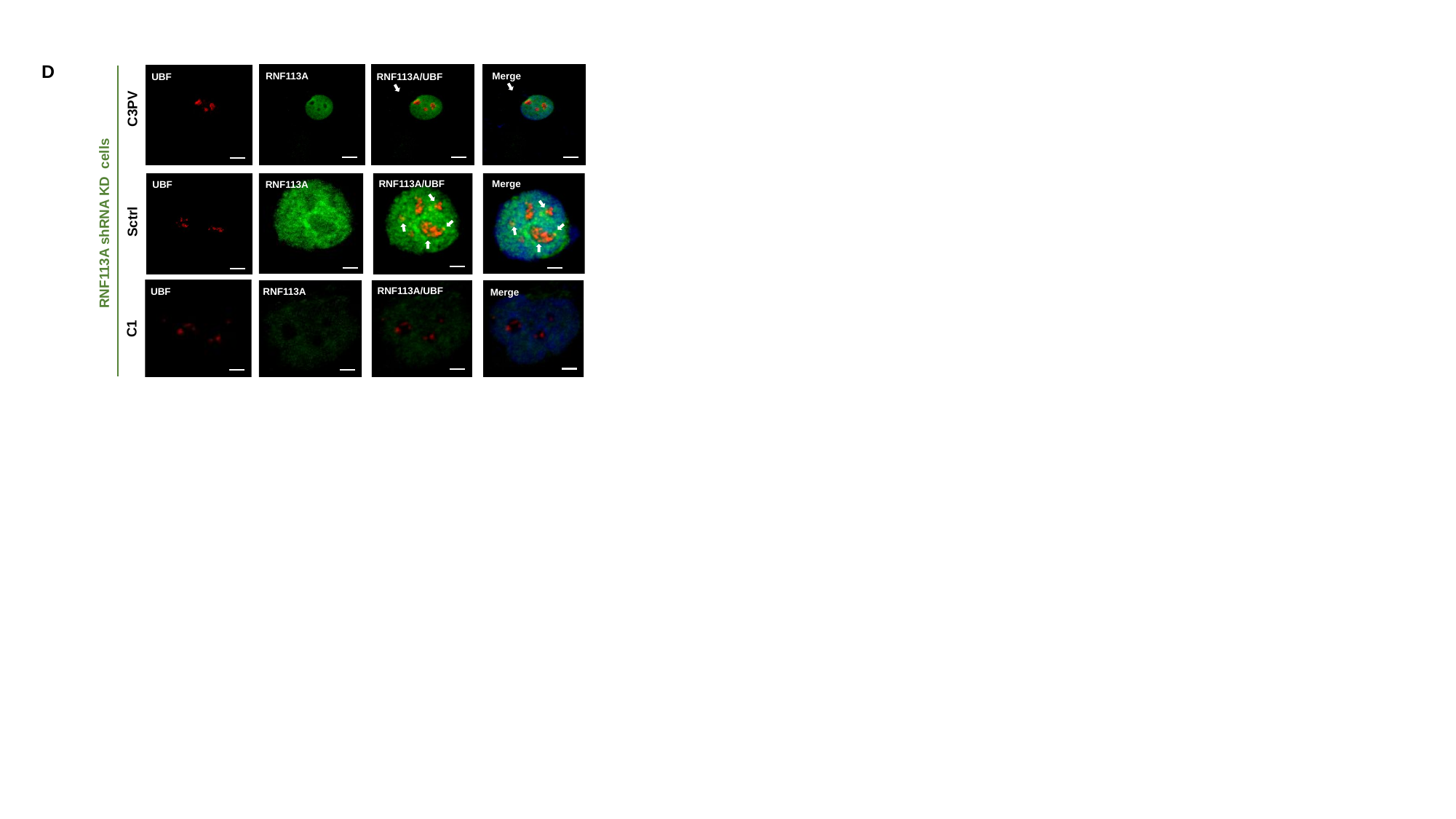

D
RNF113A
Merge
RNF113A/UBF
UBF
C3PV
RNF113A/UBF
Merge
RNF113A
UBF
Sctrl
RNF113A/UBF
UBF
RNF113A
Merge
C1
RNF113A shRNA KD cells

## Slide 7
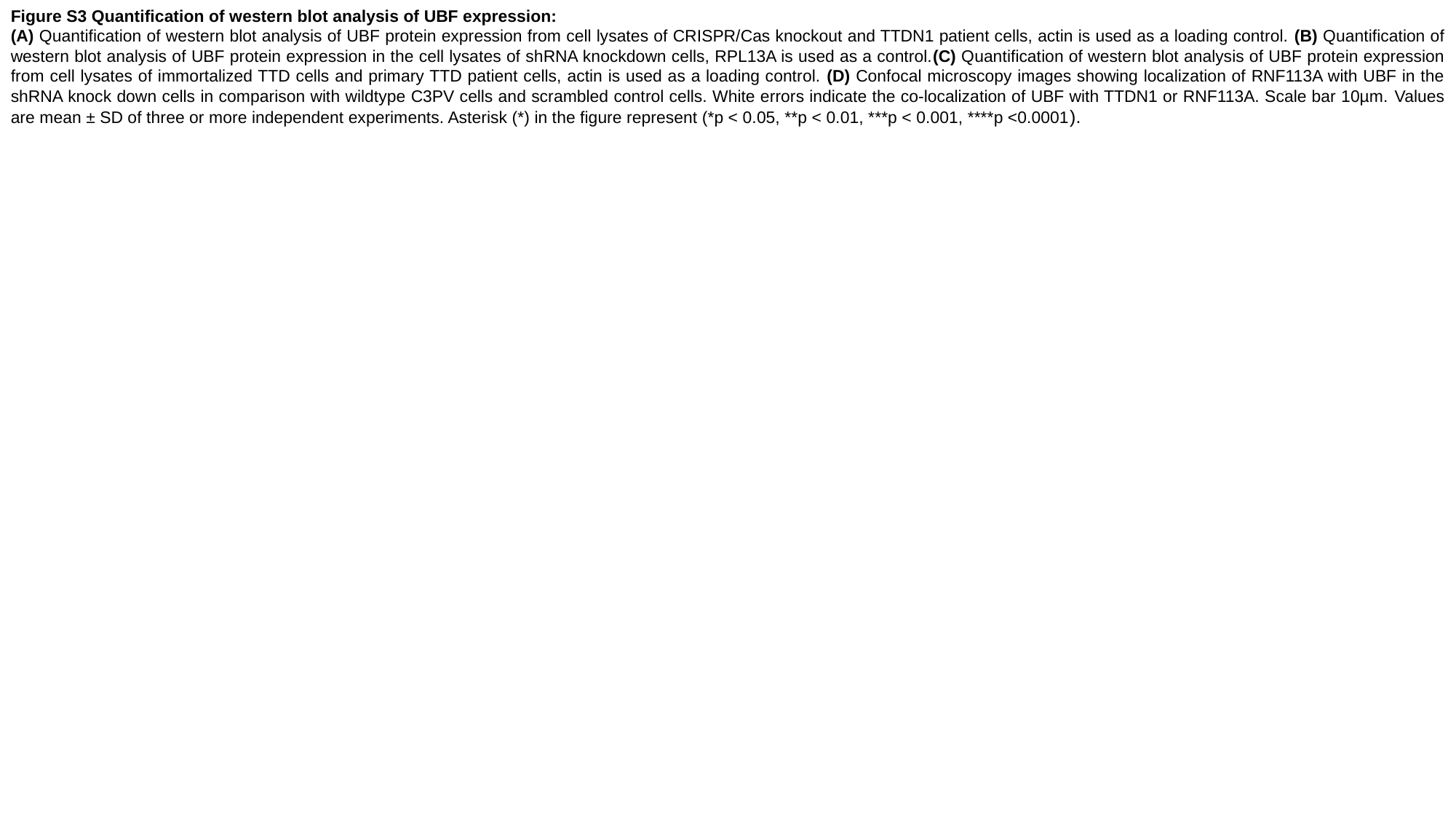

Figure S3 Quantification of western blot analysis of UBF expression:
(A) Quantification of western blot analysis of UBF protein expression from cell lysates of CRISPR/Cas knockout and TTDN1 patient cells, actin is used as a loading control. (B) Quantification of western blot analysis of UBF protein expression in the cell lysates of shRNA knockdown cells, RPL13A is used as a control.(C) Quantification of western blot analysis of UBF protein expression from cell lysates of immortalized TTD cells and primary TTD patient cells, actin is used as a loading control. (D) Confocal microscopy images showing localization of RNF113A with UBF in the shRNA knock down cells in comparison with wildtype C3PV cells and scrambled control cells. White errors indicate the co-localization of UBF with TTDN1 or RNF113A. Scale bar 10µm. Values are mean ± SD of three or more independent experiments. Asterisk (*) in the figure represent (*p < 0.05, **p < 0.01, ***p < 0.001, ****p <0.0001).

## Slide 8
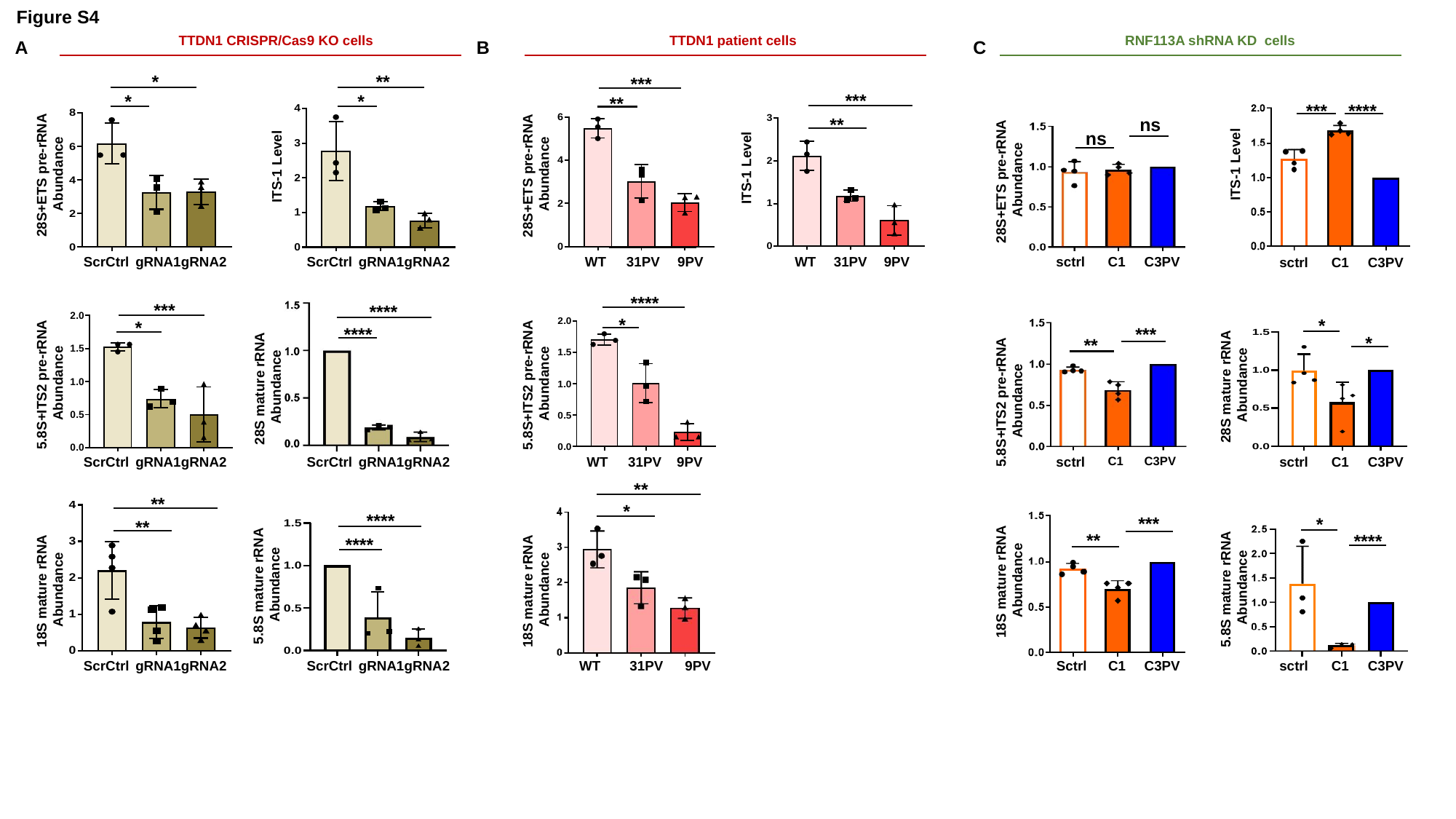

B
Figure S4
TTDN1 CRISPR/Cas9 KO cells
TTDN1 patient cells
RNF113A shRNA KD cells
A
B
C
*
*
ScrCtrl
gRNA1
gRNA2
28S+ETS pre-rRNA
Abundance
***
**
28S+ETS pre-rRNA
Abundance
WT
31PV
9PV
ns
ns
sctrl
C1
C3PV
28S+ETS pre-rRNA
Abundance
**
*
ITS-1 Level
ScrCtrl
gRNA1
gRNA2
***
**
ITS-1 Level
WT
9PV
31PV
****
***
ITS-1 Level
sctrl
C1
C3PV
***
*
ScrCtrl
gRNA1
gRNA2
5.8S+ITS2 pre-rRNA
Abundance
****
*
5.8S+ITS2 pre-rRNA
Abundance
WT
31PV
9PV
*
*
28S mature rRNA
Abundance
sctrl
C1
C3PV
****
****
28S mature rRNA
Abundance
ScrCtrl
gRNA1
gRNA2
***
**
5.8S+ITS2 pre-rRNA
Abundance
sctrl
C1
C3PV
****
****
5.8S mature rRNA
Abundance
ScrCtrl
gRNA1
gRNA2
**
*
18S mature rRNA
Abundance
WT
9PV
31PV
*
****
5.8S mature rRNA
Abundance
sctrl
C1
C3PV
**
**
18S mature rRNA
Abundance
ScrCtrl
gRNA1
gRNA2
***
**
18S mature rRNA
Abundance
Sctrl
C1
C3PV

## Slide 9
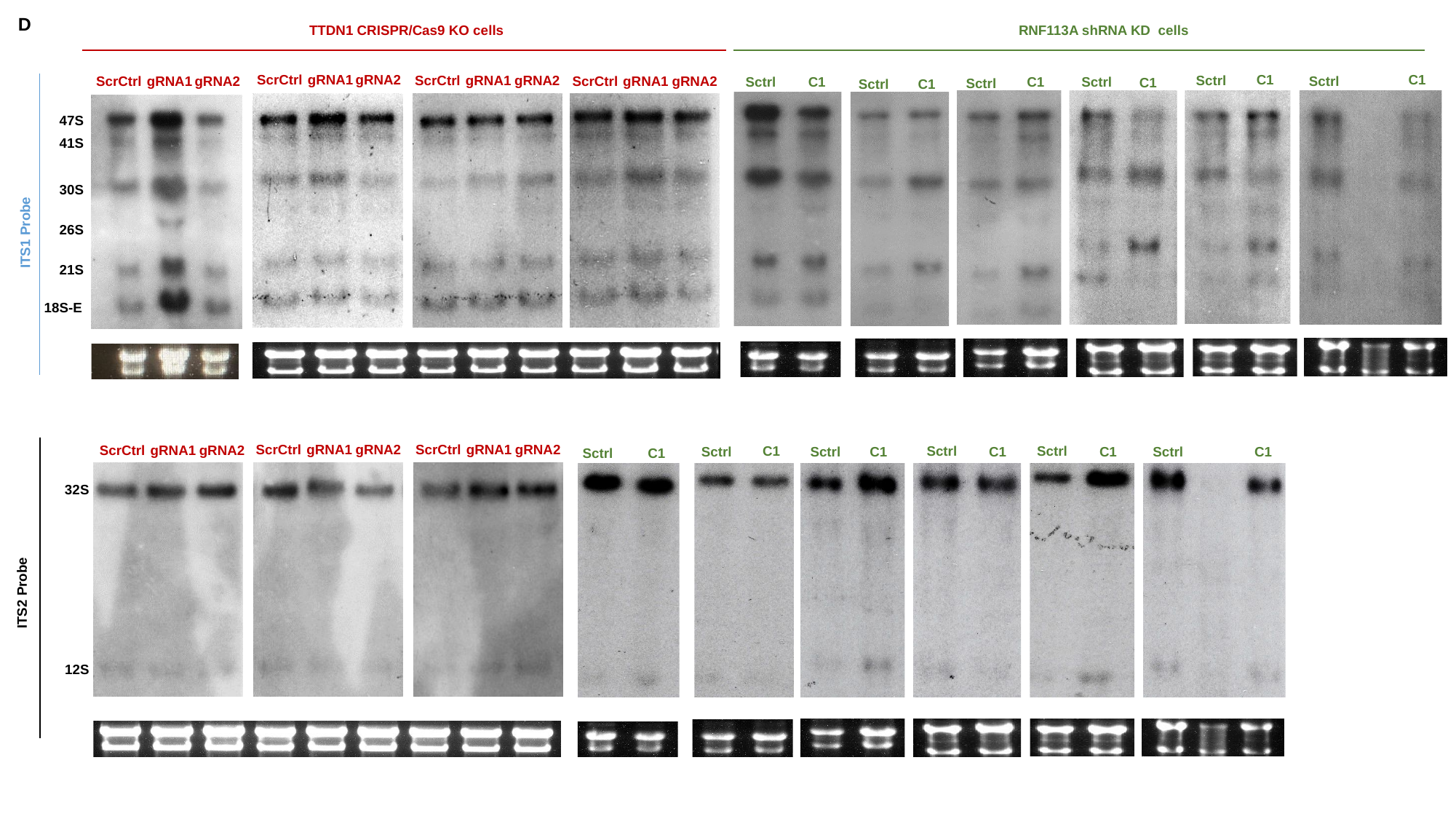

D
RNF113A shRNA KD cells
TTDN1 CRISPR/Cas9 KO cells
C1
C1
ScrCtrl
gRNA1
gRNA2
ScrCtrl
gRNA1
gRNA2
Sctrl
Sctrl
ScrCtrl
gRNA1
gRNA2
ScrCtrl
gRNA1
gRNA2
C1
Sctrl
Sctrl
C1
C1
Sctrl
Sctrl
C1
47S
41S
30S
26S
ITS1 Probe
21S
18S-E
ScrCtrl
gRNA1
gRNA2
ScrCtrl
gRNA1
gRNA2
ScrCtrl
gRNA1
gRNA2
C1
Sctrl
Sctrl
Sctrl
Sctrl
C1
C1
C1
Sctrl
C1
Sctrl
C1
32S
ITS2 Probe
12S

## Slide 10
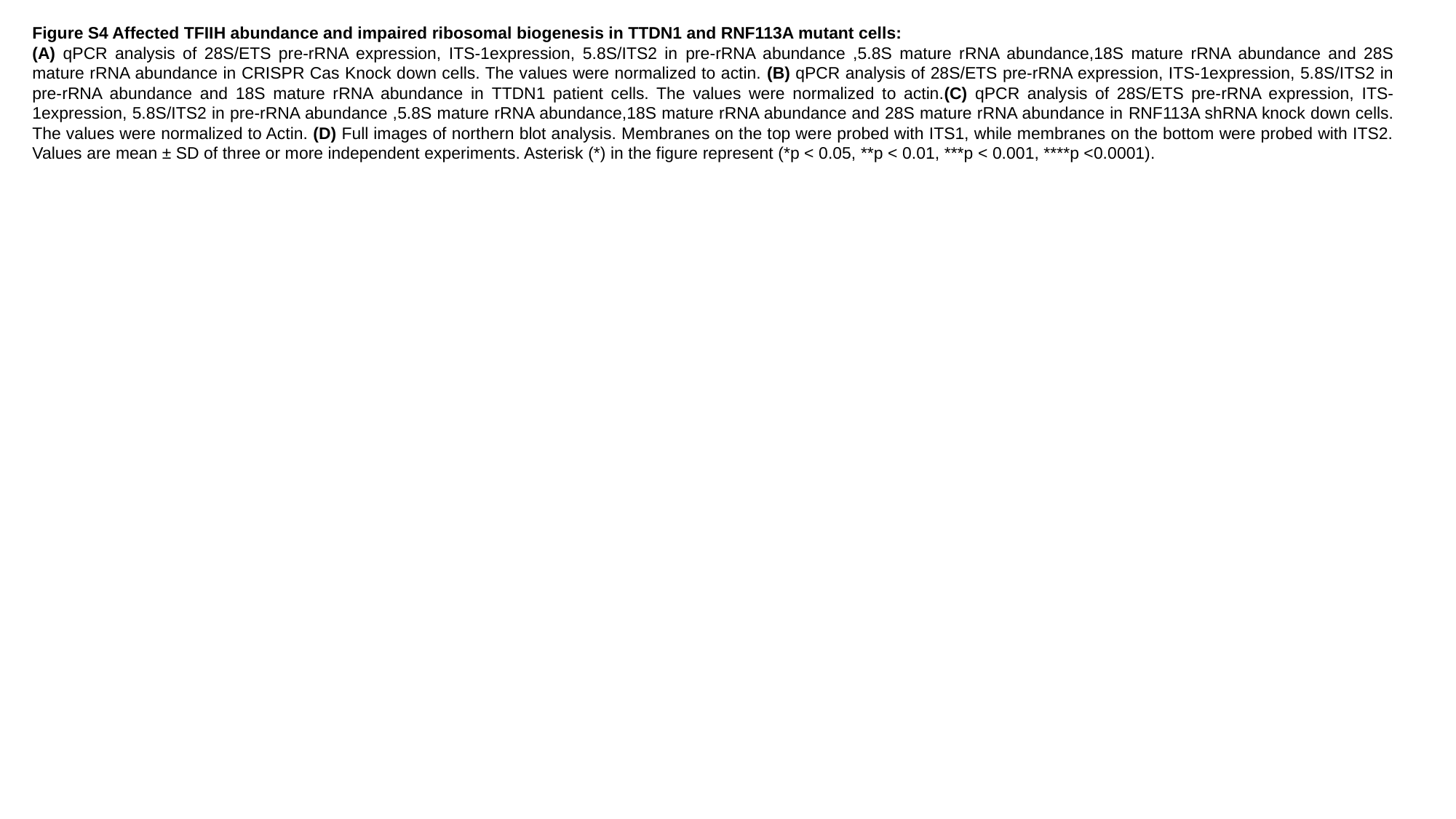

Figure S4 Affected TFIIH abundance and impaired ribosomal biogenesis in TTDN1 and RNF113A mutant cells:
(A) qPCR analysis of 28S/ETS pre-rRNA expression, ITS-1expression, 5.8S/ITS2 in pre-rRNA abundance ,5.8S mature rRNA abundance,18S mature rRNA abundance and 28S mature rRNA abundance in CRISPR Cas Knock down cells. The values were normalized to actin. (B) qPCR analysis of 28S/ETS pre-rRNA expression, ITS-1expression, 5.8S/ITS2 in pre-rRNA abundance and 18S mature rRNA abundance in TTDN1 patient cells. The values were normalized to actin.(C) qPCR analysis of 28S/ETS pre-rRNA expression, ITS-1expression, 5.8S/ITS2 in pre-rRNA abundance ,5.8S mature rRNA abundance,18S mature rRNA abundance and 28S mature rRNA abundance in RNF113A shRNA knock down cells. The values were normalized to Actin. (D) Full images of northern blot analysis. Membranes on the top were probed with ITS1, while membranes on the bottom were probed with ITS2. Values are mean ± SD of three or more independent experiments. Asterisk (*) in the figure represent (*p < 0.05, **p < 0.01, ***p < 0.001, ****p <0.0001).

## Slide 11
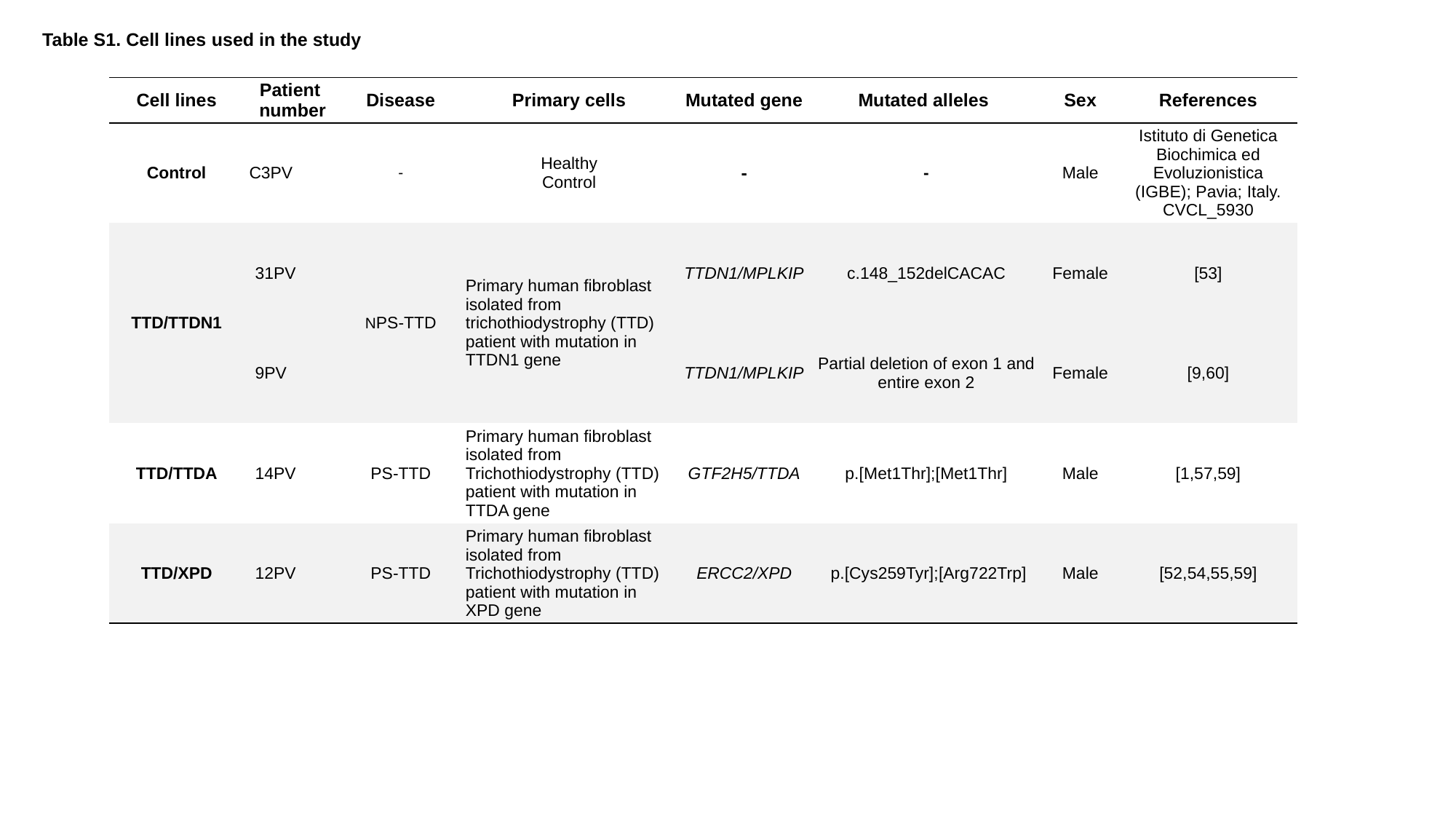

Table S1. Cell lines used in the study
| Cell lines | Patient number | Disease | Primary cells | Mutated gene | Mutated alleles | Sex | References |
| --- | --- | --- | --- | --- | --- | --- | --- |
| Control | C3PV | - | Healthy Control | - | - | Male | Istituto di Genetica Biochimica ed Evoluzionistica (IGBE); Pavia; Italy. CVCL\_5930 |
| TTD/TTDN1 | 31PV | NPS-TTD | Primary human fibroblast isolated from trichothiodystrophy (TTD) patient with mutation in TTDN1 gene | TTDN1/MPLKIP | c.148\_152delCACAC | Female | [53] |
| | 9PV | | | TTDN1/MPLKIP | Partial deletion of exon 1 and entire exon 2 | Female | [9,60] |
| TTD/TTDA | 14PV | PS-TTD | Primary human fibroblast isolated from Trichothiodystrophy (TTD) patient with mutation in TTDA gene | GTF2H5/TTDA | p.[Met1Thr];[Met1Thr] | Male | [1,57,59] |
| TTD/XPD | 12PV | PS-TTD | Primary human fibroblast isolated from Trichothiodystrophy (TTD) patient with mutation in XPD gene | ERCC2/XPD | p.[Cys259Tyr];[Arg722Trp] | Male | [52,54,55,59] |

## Slide 12
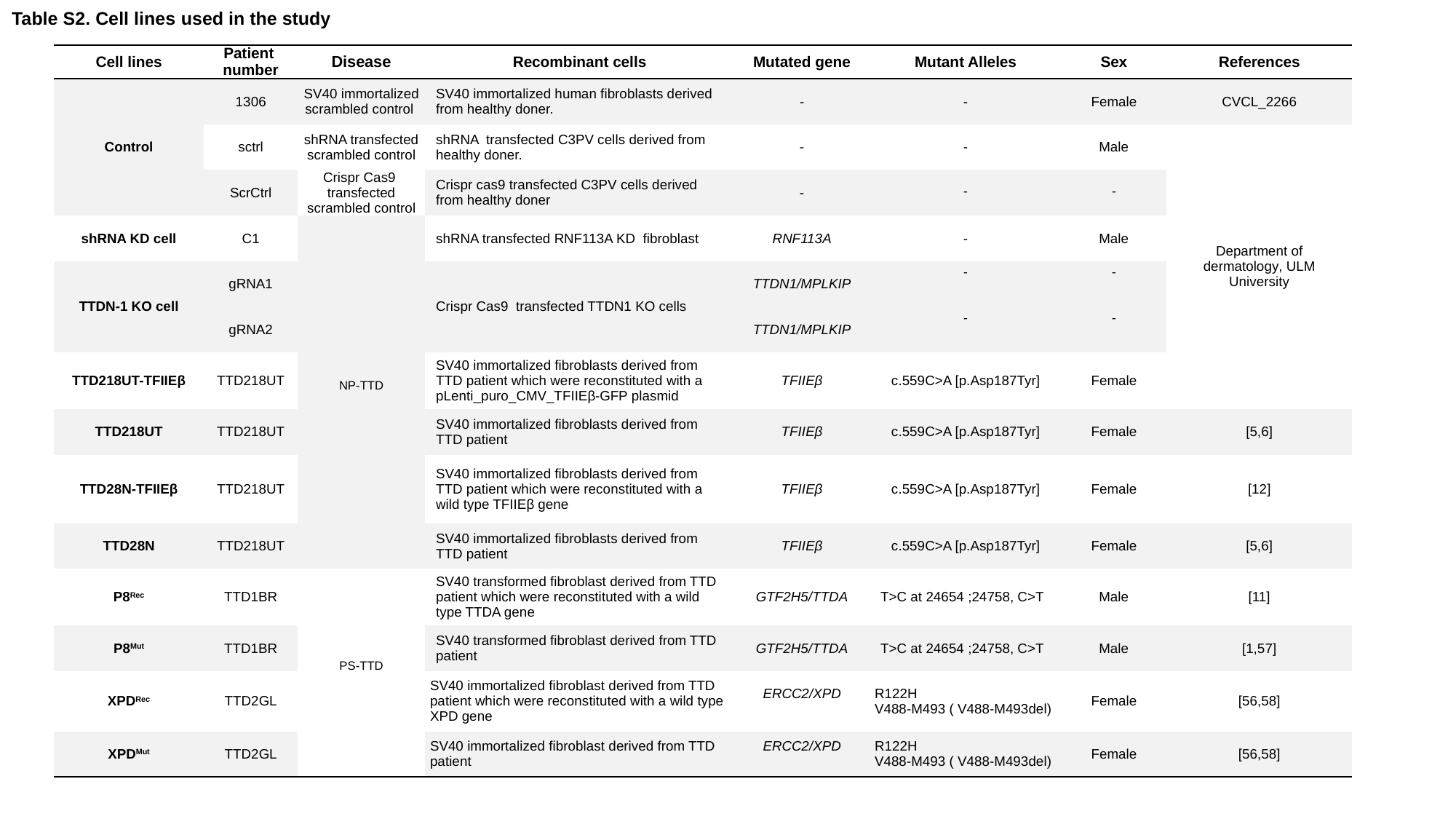

Table S2. Cell lines used in the study
| Cell lines | Patient number | Disease | Recombinant cells | Mutated gene | Mutant Alleles | Sex | References |
| --- | --- | --- | --- | --- | --- | --- | --- |
| Control | 1306 | SV40 immortalized scrambled control | SV40 immortalized human fibroblasts derived from healthy doner. | - | - | Female | CVCL\_2266 |
| | sctrl | shRNA transfected scrambled control | shRNA transfected C3PV cells derived from healthy doner. | - | - | Male | Department of dermatology, ULM University |
| | ScrCtrl | Crispr Cas9 transfected scrambled control | Crispr cas9 transfected C3PV cells derived from healthy doner | - | - | - | |
| shRNA KD cell | C1 | NP-TTD | shRNA transfected RNF113A KD fibroblast | RNF113A | - | Male | |
| TTDN-1 KO cell | gRNA1 | | Crispr Cas9 transfected TTDN1 KO cells | TTDN1/MPLKIP | - | - | |
| | gRNA2 | | | TTDN1/MPLKIP | - | - | |
| TTD218UT-TFIIEβ | TTD218UT | | SV40 immortalized fibroblasts derived from TTD patient which were reconstituted with a pLenti\_puro\_CMV\_TFIIEβ-GFP plasmid | TFIIEβ | c.559C>A [p.Asp187Tyr] | Female | |
| TTD218UT | TTD218UT | | SV40 immortalized fibroblasts derived from TTD patient | TFIIEβ | c.559C>A [p.Asp187Tyr] | Female | [5,6] |
| TTD28N-TFIIEβ | TTD218UT | | SV40 immortalized fibroblasts derived from TTD patient which were reconstituted with a wild type TFIIEβ gene | TFIIEβ | c.559C>A [p.Asp187Tyr] | Female | [12] |
| TTD28N | TTD218UT | | SV40 immortalized fibroblasts derived from TTD patient | TFIIEβ | c.559C>A [p.Asp187Tyr] | Female | [5,6] |
| P8Rec | TTD1BR | PS-TTD | SV40 transformed fibroblast derived from TTD patient which were reconstituted with a wild type TTDA gene | GTF2H5/TTDA | T>C at 24654 ;24758, C>T | Male | [11] |
| P8Mut | TTD1BR | | SV40 transformed fibroblast derived from TTD patient | GTF2H5/TTDA | T>C at 24654 ;24758, C>T | Male | [1,57] |
| XPDRec | TTD2GL | | SV40 immortalized fibroblast derived from TTD patient which were reconstituted with a wild type XPD gene | ERCC2/XPD | R122H V488-M493 ( V488-M493del) | Female | [56,58] |
| XPDMut | TTD2GL | | SV40 immortalized fibroblast derived from TTD patient | ERCC2/XPD | R122H V488-M493 ( V488-M493del) | Female | [56,58] |

## Slide 13
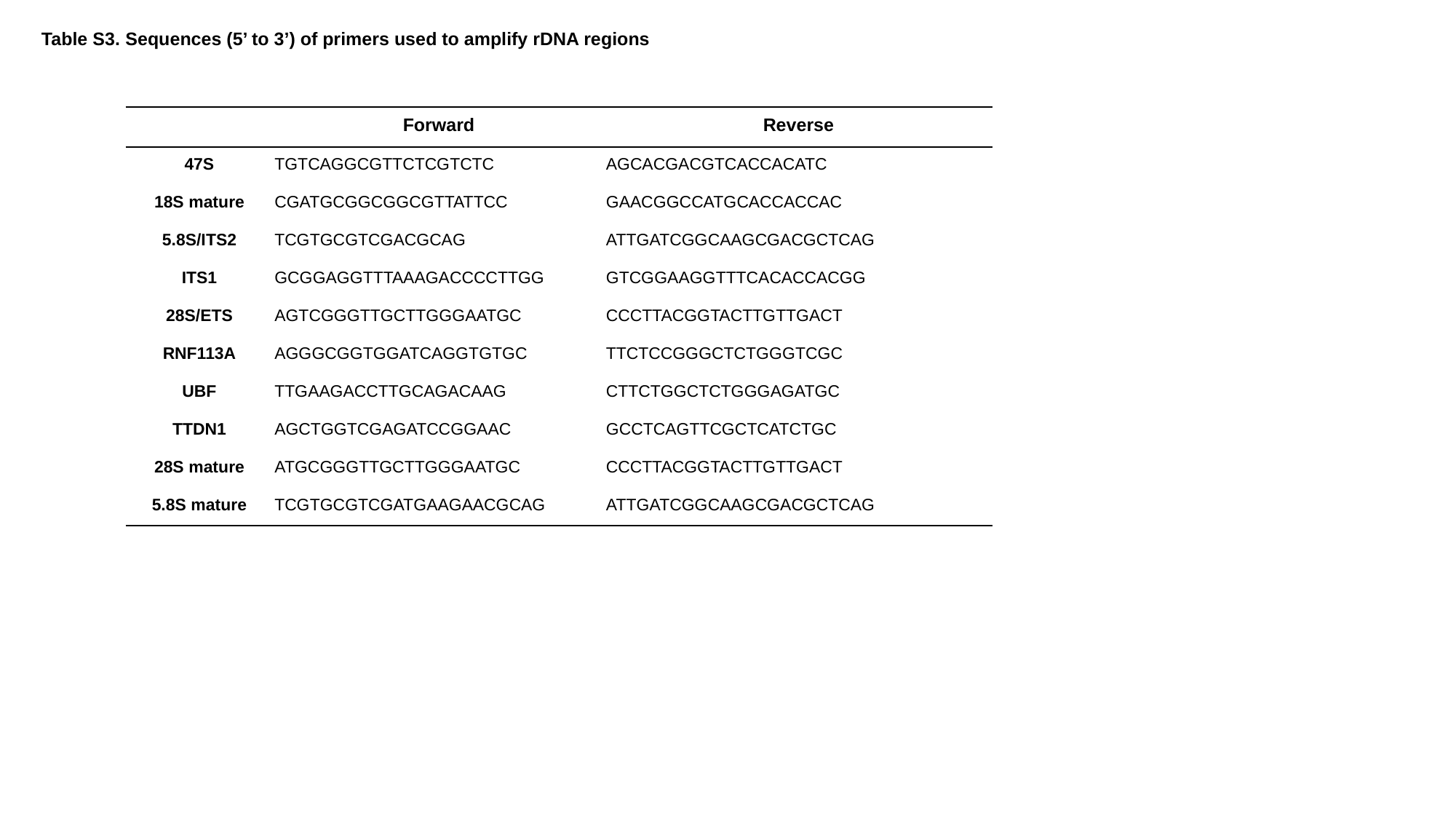

Table S3. Sequences (5’ to 3’) of primers used to amplify rDNA regions
| | Forward | Reverse |
| --- | --- | --- |
| 47S | TGTCAGGCGTTCTCGTCTC | AGCACGACGTCACCACATC |
| 18S mature | CGATGCGGCGGCGTTATTCC | GAACGGCCATGCACCACCAC |
| 5.8S/ITS2 | TCGTGCGTCGACGCAG | ATTGATCGGCAAGCGACGCTCAG |
| ITS1 | GCGGAGGTTTAAAGACCCCTTGG | GTCGGAAGGTTTCACACCACGG |
| 28S/ETS | AGTCGGGTTGCTTGGGAATGC | CCCTTACGGTACTTGTTGACT |
| RNF113A | AGGGCGGTGGATCAGGTGTGC | TTCTCCGGGCTCTGGGTCGC |
| UBF | TTGAAGACCTTGCAGACAAG | CTTCTGGCTCTGGGAGATGC |
| TTDN1 | AGCTGGTCGAGATCCGGAAC | GCCTCAGTTCGCTCATCTGC |
| 28S mature | ATGCGGGTTGCTTGGGAATGC | CCCTTACGGTACTTGTTGACT |
| 5.8S mature | TCGTGCGTCGATGAAGAACGCAG | ATTGATCGGCAAGCGACGCTCAG |

## Slide 14
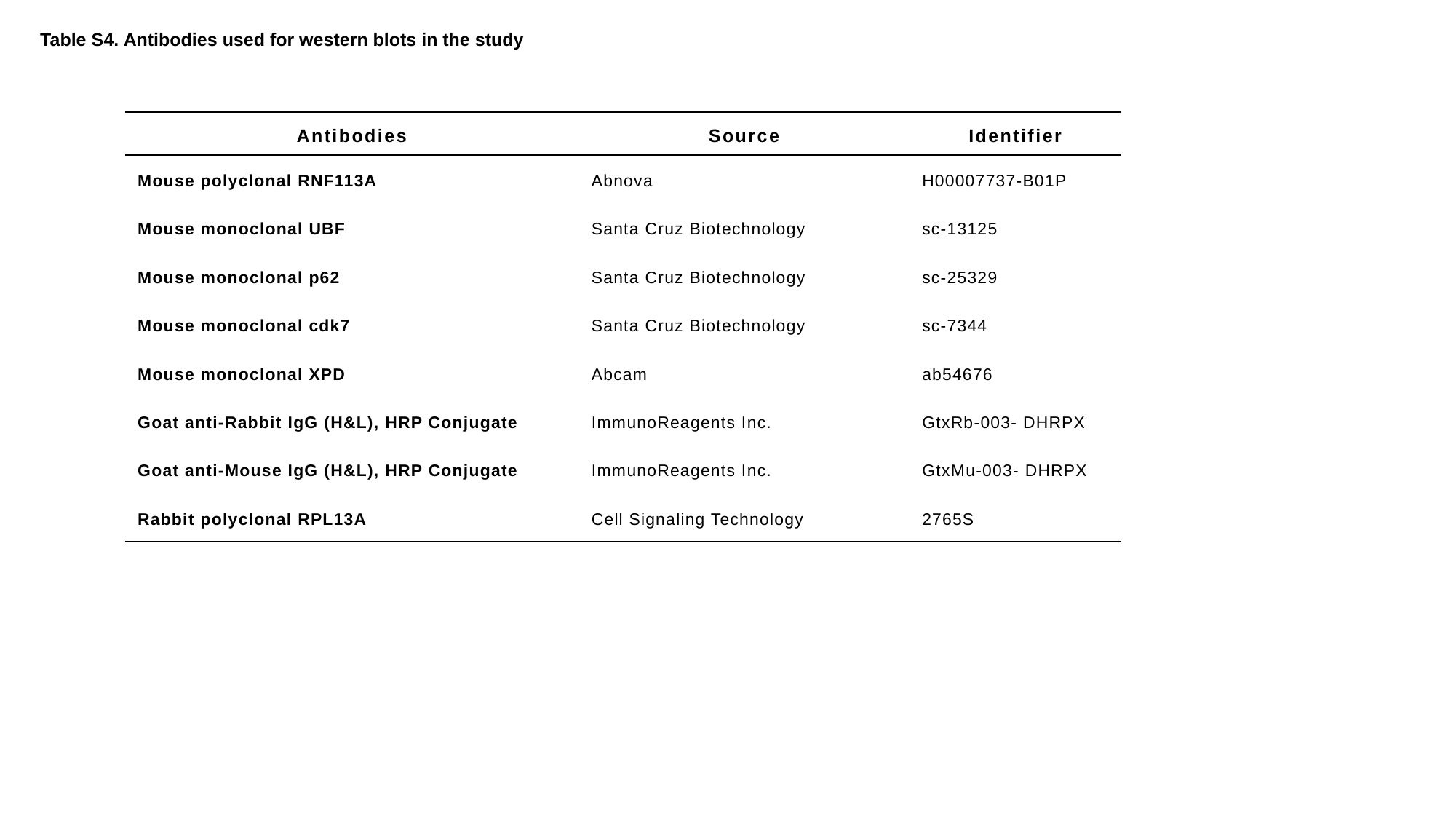

Table S4. Antibodies used for western blots in the study
| Antibodies | Source | Identifier |
| --- | --- | --- |
| Mouse polyclonal RNF113A | Abnova | H00007737-B01P |
| Mouse monoclonal UBF | Santa Cruz Biotechnology | sc-13125 |
| Mouse monoclonal p62 | Santa Cruz Biotechnology | sc-25329 |
| Mouse monoclonal cdk7 | Santa Cruz Biotechnology | sc-7344 |
| Mouse monoclonal XPD | Abcam | ab54676 |
| Goat anti-Rabbit IgG (H&L), HRP Conjugate | ImmunoReagents Inc. | GtxRb-003- DHRPX |
| Goat anti-Mouse IgG (H&L), HRP Conjugate | ImmunoReagents Inc. | GtxMu-003- DHRPX |
| Rabbit polyclonal RPL13A | Cell Signaling Technology | 2765S |

## Slide 15
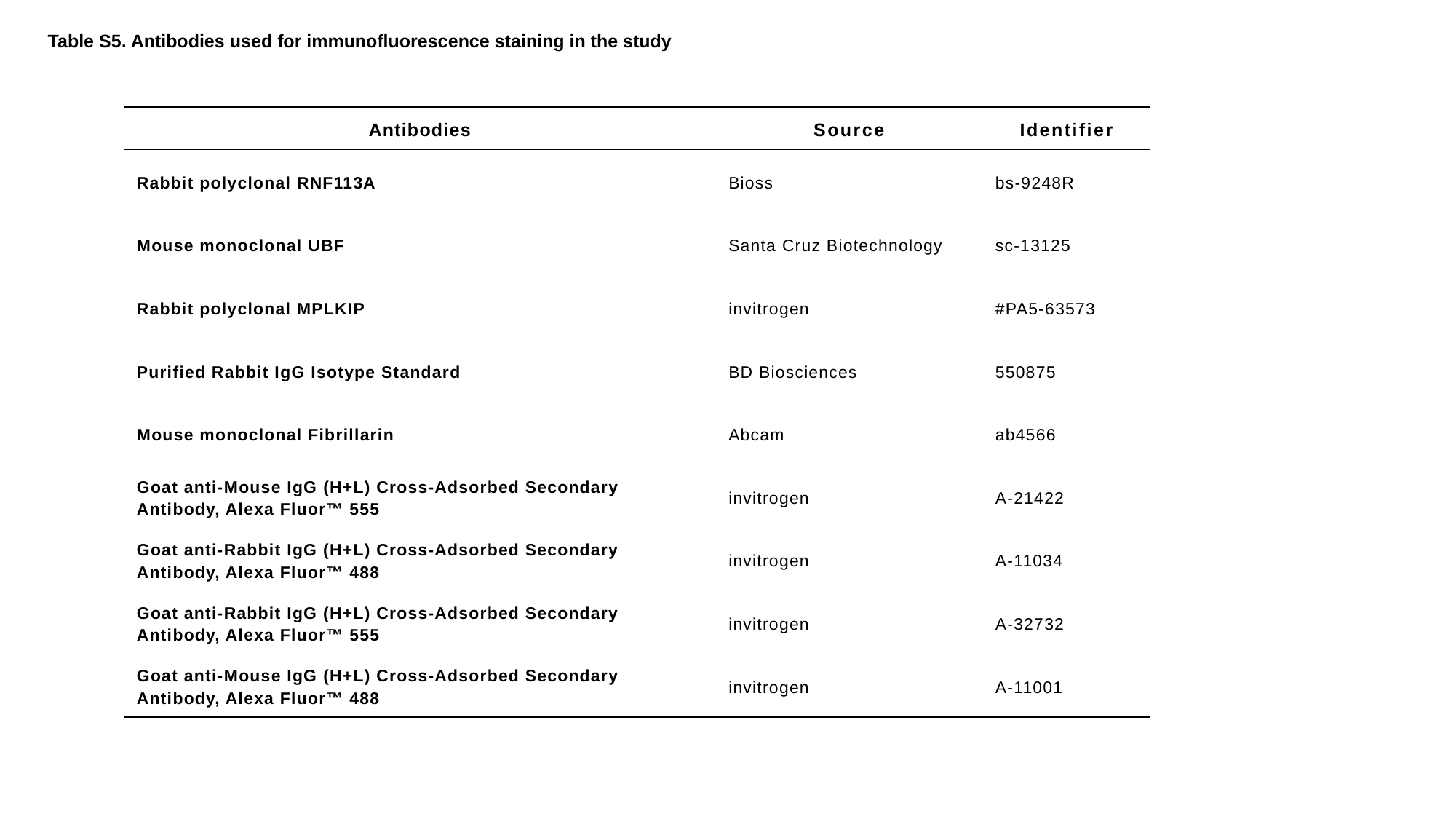

Table S5. Antibodies used for immunofluorescence staining in the study
| Antibodies | Source | Identifier |
| --- | --- | --- |
| Rabbit polyclonal RNF113A | Bioss | bs-9248R |
| Mouse monoclonal UBF | Santa Cruz Biotechnology | sc-13125 |
| Rabbit polyclonal MPLKIP | invitrogen | #PA5-63573 |
| Purified Rabbit IgG Isotype Standard | BD Biosciences | 550875 |
| Mouse monoclonal Fibrillarin | Abcam | ab4566 |
| Goat anti-Mouse IgG (H+L) Cross-Adsorbed Secondary Antibody, Alexa Fluor™ 555 | invitrogen | A-21422 |
| Goat anti-Rabbit IgG (H+L) Cross-Adsorbed Secondary Antibody, Alexa Fluor™ 488 | invitrogen | A-11034 |
| Goat anti-Rabbit IgG (H+L) Cross-Adsorbed Secondary Antibody, Alexa Fluor™ 555 | invitrogen | A-32732 |
| Goat anti-Mouse IgG (H+L) Cross-Adsorbed Secondary Antibody, Alexa Fluor™ 488 | invitrogen | A-11001 |
